# Current status of establishing a venous line in CPA patients by Emergency Life-Saving Technicians in the prehospital setting in Japan and a proposal for intraosseous infusion

**DOI:** 10.1186/1865-1380-5-2

**Published:** 2012-01-09

**Authors:** Kenji Isayama, Toshio Nakatani, Masanobu Tsuda, Akihiko Hirakawa

**Affiliations:** 1 Department of Emergency and Critical Care Medicine, Kansai Medical University, 10-15, Fumizonocho, Moriguchi, Osaka, 570-8507, Japan; 2 Department of Emergency and Critical Care Medicine, Fujita Health University, Aichi, Japan

## Abstract

**Introduction:**

It is important to have a venous line in cardiopulmonary arrest (CPA) patients as an emergency treatment measure in prehospital settings, but establishment of a peripheral venous line is difficult in such patients. This study aimed to investigate the current status of intravenous infusion (IVI) in CPA patients by Emergency Life-Saving Technicians (ELSTs) in Japan. We also considered alternative measures in case IVI was difficult or impossible.

**Methods:**

We investigated a nationwide database between 1 January 2005 and 31 December 2008. From a total of 431,968 CPA cases, we calculated the IVI success rate and related parameters.

The Bone Injection Gun (BIG) and simulator legs (adult, pediatric, and infant) were used by 100 ELSTs selected for the study to measure the time required and the success rate for intraosseous infusion (IOI).

**Results:**

The number of CPA patients, IVI, adrenaline administration, and the IVI success rate in adult CPA patients increased every year. However, the IVI success rate in pediatric CPA patients did not increase. Although adrenaline administration elevated the ROSC rate, there was no improvement in the 1-month survival rate. The time required for IOI with BIG was not different among the leg models. The success rates of IOI with BIG were 93%, 94%, and 84% (*p *< 0.05 vs. adult and pediatric) in adult, pediatric, and infant models, respectively.

**Conclusions:**

The rate of success of IVI in adult CPA patients has been increased yearly in Japan. However, as establishing a peripheral venous line in pediatric patients (1-7 years old) by ELSTs is extremely difficult in prehospital settings, there was no increase in the IVI success rate in such patients. As the study findings indicated IOI with BIG was easy and rapid, it may be necessary to consider IOI with BIG as an alternative option in case IVI is difficult or impossible in adult and pediatric patients.

## Introduction

The Emergency Life-Saving Technicians (ELSTs) system was established in Japan in 1991 as one of the emergency medical service (EMS) systems. ELSTs are permitted to perform endotracheal intubation, intravenous infusion (IVI) of Ringer's lactate solution, and adrenaline administration through a venous line. However, these treatments are allowed only for cardiopulmonary arrest (CPA) patients. Hence, in Japan prehospital care activities of ELSTs are very limited compared with those in western countries [1-4]. ELSTs are not permitted to perform advanced life support (ALS) such as needle thoracostomy, blood glucose measurements to differentiate hypoglycemic coma, administration of medications other than adrenaline, and intraosseous infusion (IOI) instead of IVI.

Introduction of the Utstein style template enabled the evaluation and comparison of national, regional, and hospital based EMS systems worldwide [5-7]. In January 2005, the Fire and Disaster Management Agency (FDMA) of Japan began accumulating data for out-of-hospital cardiac arrest patients using the Utstein template [8].

Among treatments for CPA patients, although the effects of defibrillation, administration of adrenalin, and chest compression have been reported in detail using the Utstein template [9,10], the current status and the effects of IVI by ELSTs have not been reported from Japanese nationwide analysis. Therefore, we focused this study on the analysis of IVI using large-scale data of the Utstein template in Japan.

It is important to have a venous line in CPA patients as an emergency treatment in prehospital settings, but establishment of a peripheral venous line is difficult, especially in dehydrated or hemodynamically unstable patients, particularly so because their peripheral blood vessels are frequently collapsed [11,12]. In Japan, among the medical techniques permitted for ELSTs, establishment of a venous line is less frequently attempted and is less successful compared to airway management with devices [13]. Establishing a venous line is essential to administer medications for patients in a prehospital setting. Unfortunately, in many CPA cases that KI encountered as an ELST, it was difficult to establish a peripheral venous line in prehospital settings. Availability of a venous line on arrival at the hospital is helpful for immediate administration of medications and fluids [14]. Obtaining rapid and reliable vascular access is also crucial for the prompt care of critically ill children and adults [15].

However, if it is impossible to perform an immediate IVI in patients, IOI may be an excellent alternative for providing vascular access to administer medications and fluids. Recently, mechanical IOI devices have become more convenient to use compared to manual IOI devices [16,17]. The Bone Injection Gun ™ (BIG, WaisMed Ltd., Hertzeliya, Israel) is a small semi-automatic, disposable, spring-loaded IOI device with a trigger. The BIG was the only mechanical IOI device approved in Japan by 2008. It has been reported that the use of the BIG results in rapid and easy administration of IOI medications and fluids for adults and children with good results [15,18].

The purpose of this study is to investigate the current status of IVI in CPA patients by ELSTs in Japan. Furthermore, we examine the usefulness of IOI with BIG by ELSTs as an alternative option in case IVI is extremely difficult or impossible.

## Methods

### Study design (the Utstein style database)

We investigated a nationwide database for all patients throughout Japan who were transported to hospitals with CPA by ambulances from all Japanese Fire Departments. A total of 431,968 patient records were collected prospectively and accumulated by the FDMA using the Utstein template between 1 January 2005 and 31 December 2008. Some results of this study, such as the rate of return of spontaneous circulation (ROSC) with defibrillation and chest compression, have been reported elsewhere [8,10]. We, therefore, focused on the success rate for establishing a venous line by ELSTs. We also compared the rate of ROSC and the 1-month survival rate with or without adrenaline administration by ELSTs in 2008.

In this study, we defined pediatric cases as children between 1-15 years of age. All CPA patients were categorized in age brackets. We also divided pediatric cases into two groups, 1-7 and 8-14 years of age, because adrenaline administration is only indicated for the 8-14-year-old group in Japan.

### Study design (IOI using BIG)

We measured the time required and the success rate for IOI by ELSTs using training BIG and simulator legs (adult, pediatric, and infant). ELSTs tried IOI with BIG in the adult, pediatric, and infant models respectively in spacious surroundings.

#### Study participants

In this study, 100 active volunteer ELSTs selected conveniently from 11 fire department headquarters in Japan participated. They had never used BIG previously.

#### Study instruments

In this study, we used the adult training BIG (15G, WaisMed Ltd., WMBIG-DEMO-A1, Hertzelia, Israel) and the pediatric training BIG (18G, WaisMed Ltd., WMBIG-DEMO-C2, Hertzelia, Israel). Instruction for usage is the same as for the actual BIG.

#### IOI model and penetration site

Three lower leg models were used in this study. The adult model was made by us. To enable training in BIG needle use, a round hole was cut in the tibial plateau of a mannequin's leg. A small reservoir was fitted into this hole and covered with an artificial bonelike material and silicone rubber "skin." The pediatric model was the lower leg of the Megacode Kid CPR-7500 (no. 231-05050, Laerdal Medical AS, Stavanger, Norway). The Megacode Kid is a full-body mannequin reproduction of a 6-year-old boy that is designed for simulation and enables IOI on a pediatric model. The infant model was the lower leg of the ALS baby trainer (no. 08003005, Laerdal Medical AS, Stavanger, Norway). The ALS baby trainer represents a 3-month-old, 5-kg baby and is a simulator designed to provide IOI on an infant model.

The penetration site of the adult leg model was the point two fingerbreadths inside and one fingerbreadth cranial from the tibial tuberosity. The penetration site of pediatric and infant leg models was the point one fingerbreadth inside and one fingerbreadth caudal from the tibial tuberosity.

#### Study procedure

Before commencement of the trial, ELSTs received a brief explanation of a standardized BIG procedure and observed the BIG demonstration by the author. Thereafter, ELSTs practiced once with each model. We measured the time required from selection of the insert point until connecting an infusion line wearing rubber gloves. Successful insertion was defined as a bare needle anchored in a firm upright position in the penetration site.

#### Questionnaire survey

ELSTs participating in this study were asked their opinion of whether the device was user friendly, easy to learn, simple, easy to use, and safe to use, and about appropriateness of the BIG for their work environment.

### Statistical analysis

Data were calculated and analyzed using Microsoft Excel 2007 (Microsoft Corp., Redmond, WA). All values were shown with mean ± standard error of the mean (SEM). Chi-square tests were used to analyze the rate of ROSC, the 1-month rate of survival, and the success rates of each simulator model for IOI with BIG. The time required of each simulator model for IOI with BIG was analyzed according to one-way analysis of variance (ANOVA) followed by Fisher's PLSD (Fisher's Protected Least Significant Difference). The significance level was set at *p *< 0.05.

## Results

### Analysis of the database

#### Numbers and rates of IVI success

**Figure 1:**
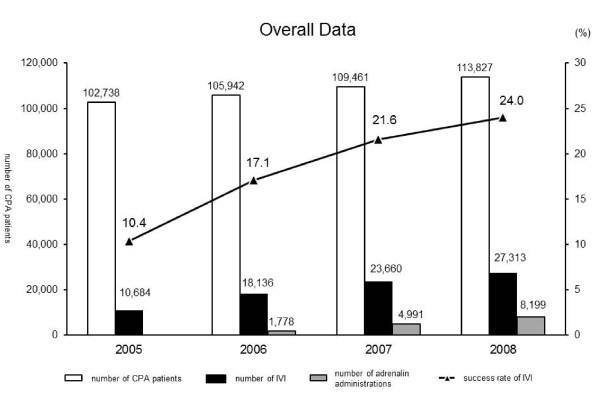
**Overall data on out-of hospital CPA patients in 2005-2008**. Number of CPA patients shows all patients throughout Japan who were transported to the hospital under CPA by ambulances of Japanese Fire Departments in a 1-year period. Number of IVIs indicates the number of successful IVIs in CPA patients by ELSTs. Number of adrenaline administrations indicates the number of successful adrenaline administrations after IVI in CPA patients by ELSTs. The success rate of IVI (%) was calculated as the number of IVIs divided by the number of CPA patients.

**Figure 2:**
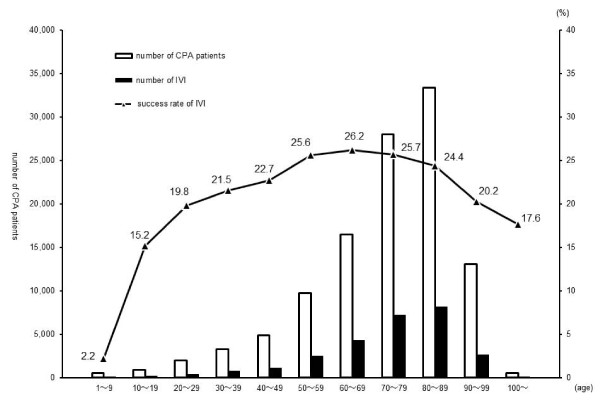
**Comparison of the number of IVIs and success rate in CPA patients in 2008**. Number of CPA patients shows all patients throughout Japan who were transported to the hospital under CPA by ambulances of Japanese Fire Departments in 2008. Number of IVIs shows successful IVIs established by ELSTs in CPA patients in 2008. The success rate of IVI (%) was calculated as the number of IVIs divided by the number of CPA patients in age brackets in 2008.

**Figure 3:**
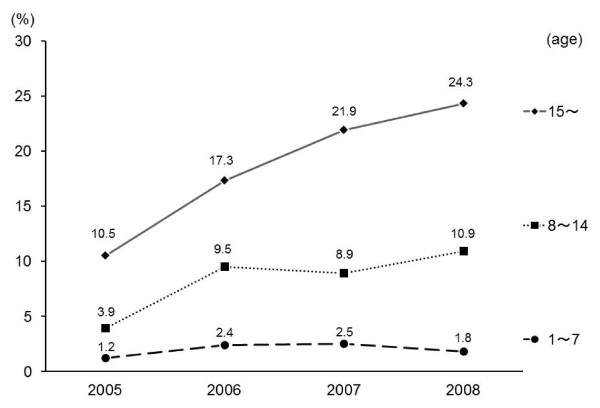
**The success rates of IVI for age groups in 2005-2008**. The success rates of IVI in CPA patients in age groups 1-7, 8-14, and above 15 in 2005-2008.

Figure 1 shows the numbers of CPA patients, IVI, adrenaline administration, and the rate of successful IVI in the years between 2005 and 2008. The numbers of CPA patients, IVI, adrenaline administration, and the success rate of IVI all increased from 2005 to 2008. Figure 2 compares the numbers of CPA patients, IVI, and the success rate of IVI in all age brackets in 2008. The success rate of IVI in the pediatric age group (1-9 years old) was extremely low at 2.2%. Figure 3 shows the success rate of IVI in CPA patients aged 1-7, 8-14, and above 15 years from 2005 to 2008. Although the success rate of IVI in patients above 15 years of age increased every year, that in pediatric patients (8-14 years old) barely increased from 2005 to 2008. The success rate of IVI in pediatric patients (1-7 years old) did not increase, and surprisingly slightly decreased in 2008.

#### The effect of adrenaline administration

Table 1 shows the rate of ROSC and 1-month survival rate in CPA patients with or without adrenaline administration by ELSTs in 2008. The rate of ROSC was significantly higher in the group with adrenaline administration compared to the group without adrenaline administration (*p *< 0.001); however, there was no difference in the 1-month survival rate (*p *= 0.94).

**Table 1:** Return of spontaneous circulation (ROSC) and 1-month survival rate after CPA with or without adrenaline administration

	ROSC (n = 8,136)	Non ROSC (n = 104,302)	1-month survival rate after CPA (n = 5,907)	Non survival (n = 106,538)
**Adrenaline administration**	1,570 (18.5%)***	6,651 (81.5%)	427 (5.2%)	7,737 (94.8%)

**No adrenaline administration**	6,566 (6.3%)	97,691 (93.7%)	5,480 (5.3%)	98,801 (94.7%)

ROSC and 1-month survival rate after CPA for adrenaline administration were compared using the chi-square test.

The rate of ROSC was significantly higher in the group with adrenaline administration compared to the group without adrenaline administration; however, there was no difference in the 1-month survival rate. ***p < 0.001 in chi-square test.

### BIG study

#### Experimental data

The BIG study group consisted of 100 participants (volunteer ELSTs) with a mean age of 34.7 ± 0.64 years and time in career of 5.2 ± 0.28 years.

Table 2 shows the time required and the success rates for IOI with BIG in adult, pediatric, and infant leg models. The time required for performing IOI with BIG among the different leg models was similar. There was a significant difference in the success rate of IOI with BIG in infant leg models when compared to adult (*p *< 0.04) and pediatric (*p *< 0.03) leg models, according to chi-square tests. Sixteen failures occurred in 100 attempts at BIG placement in the infant model.

**Table 2:** Time required and success rates of IOI with BIG

	Time required Mean ± SEM (sec)	Success rate
**Adult**	29.1 ± 0.63	93% (93/100)

**Pediatric**	28.7 ± 0.60	94% (94/100)

**Infant**	29.3 ± 0.65	84% (84/100)*

The time required for IOI with BIG was compared using one-way analysis of variance (ANOVA) followed by Fisher's PLSD (Fisher's Protected Least Significant Difference). The success rates of IOI with BIG were compared using the chi-square test.

There was no difference in the time required for performing IOI with BIG among the different leg models. The success rates of IOI with BIG were significantly lower in infant leg models when compared to adult and pediatric leg models.

*p < 0.05 in chi-square test.

#### Questionnaire survey

The questionnaire survey revealed that ELSTs considered the BIG easy to learn and easy to place. Overall, the BIG was described as satisfactory by 90% of study participants. All participants expressed great satisfaction with IOI using the BIG, particularly in cases with difficult IVI.

## Discussion

IVI is necessary for fluid infusion and medication administration in acutely affected patients as an emergency treatment in prehospital settings [15]. However, it is not easy to establish a peripheral venous line for various reasons. In the prehospital setting, ELSTs may face additional obstacles, such as expediting patient transport [15]. Usually a hostile environment (inadequate light; a noisy, narrow space; moving ambulance; etc.) makes the introduction of IVI even more difficult. Vascular collapse or inadequate cardiac output may impair access to the peripheral vascular system, and thus hamper emergency medication and fluid administration [19].

Failure rates for IVI in the emergency setting are described as between 10%-40% [19]. The average time needed for IVI is reportedly between 2.5-16 min in patients with difficult IVI [20]. Delays in IVI in the field might be followed by additional delay in the emergency department when reattempting IVI [21]. The resultant time lag for necessary diagnostic and treatment procedures potentially compromises the patient [22]. Prompt transport of CPA patients should not be delayed solely to obtain IVI. IVI should be performed immediately during hospital transport or in a prehospital setting.

The success rate of IVI in CPA patients by ELSTs has increased yearly since authorization of adrenaline administration by ELST in 2006 (Figure 1). However, the extremely low rate of successful IVI in patients aged less than 10 years indicates that IVI is more difficult in pediatric CPA patients than in adults (Figure 2). For example, in 66 pediatric CPA patients, Rosetti et al. demonstrated that experienced emergency department personnel required more than 10 min to gain IVI in 24% of the cases; IVI was never obtained in 6% of victims [23]. As the success rate of IVI in 1-7-year-old CPA patients did not increase during the study period, this suggests that the rate may not increase in the future (Figure 3). In the expected chaotic early phases of primary resuscitation, timely IVI may be difficult or even impossible in pediatric CPA patients for inexperienced ELSTs. It may be extremely difficult to improve their skills readily for performing IVI in pediatric patients. However, it is necessary to improve IVI rates in pediatric CPA patients.

Current guidelines recommend that IOI should be established in both pediatric and adult emergency patients if it is difficult or impossible to perform an immediate IVI for CPR [24]. The American Heart Association recommends the use of IOI in patients under 6 years of age in need of vascular access who have had two failed IVI attempts or where more than 2 min have elapsed when attempting IVI [25]. Studies have shown that successful IOI within 1-2 min was possible in 72-100% of patients in the field [15,26]. Other studies have demonstrated that IOI can decrease the time needed to perform IVI in pediatric patients under CPA [25,26]. IOI of medications achieves adequate plasma concentrations in a time comparable with infusion through central and peripheral intravenous routes for all emergency medications [27,28]. However, IOI using a conventional manual IOI needle might be difficult to perform during resuscitation [29].

Mechanical IOI devices have been developed and already have been introduced in many countries; they are an excellent option. The BIG is used in battlefield and prehospital settings to easily and rapidly enable IOI in the USA and Israel [15]. Similarly, in our previous study, IOI with BIG was quick, simple, and unaffected by inexperience or difficult situations for IVI [30]. In addition, in this study, IOI with BIG was easy and rapid (Table 2). In Japan, physicians have recently started to use the BIG in several critical care medical centers and in prehospital settings, such as mobile intensive care units and helicopter emergency medical services.

The results of this study, such as the rate of ROSC with defibrillation and chest compression, have been reported elsewhere [8,10]. Compared with patients who received advanced cardiac life support without intravenous medicine administration following cardiac arrest, patients with IVI and medicine administration had a high rate of ROSC but no significant improvement in long-term survival rate [31]. Similarly, in this study, although the adrenaline administration increased the rate of ROSC, there was no difference in the 1-month survival rate (Table 1). However, in Japan the rate of successful IVI in adult CPA patients by ELSTs is low compared to western countries, particularly in pediatric CPA patients where the rate is even lower, and the rate of adrenaline administration is considerably lower (Figure 1). Therefore, we suggest that, first, it is necessary to improve the rate of successful IVI and adrenaline administrations, and subsequently, it should be considered if adrenaline administration is effective or not in CPA patients in prehospital settings in Japan (Figure 1 and Table 1).

IVI in pre-CPA patients is an emergency treatment given by ELSTs. However, for ELSTs, it is difficult to perform IVI in pre-CPA or profound shock patients because of peripheral vein collapse. IOI with BIG may be effective especially in cases where IVI is very difficult or impossible such as in pediatric CPA patients or pre-CPA patients.

It is reported that the success rate of IOI was 25% in children aged less than 1 year, 100% in children aged 1-2 years, 86% in children 3-9 years old, and 74% in patients above 10 years of age [32]. The BIG may be effective in both adults and children, except for children aged 0-11 months [32]. In this study, the success rate of IOI with BIG in the infant leg model was significantly lower compared to adult and pediatric model legs (Table 2). Because the penetration site in an infant leg is particularly small and narrow, IOI with BIG in infants would be more difficult than in adult and pediatric patients. In fact more failures occurred in BIG placement in the infant leg model. Therefore, close attention should be paid to IOI with BIG in infants.

Our study has certain limitations. We did not compare the BIG to other mechanical devices or manual IOI needles. As the model legs we used in this study are not actual human legs, this experimental data may not similarly reflect the situation for an actual human leg in emergency cases. However, results of our study at least would indicate the definite usefulness of IOI with BIG by ELSTs, particularly in those with difficult IVI conditions.

We suggest that it is necessary to consider IOI with BIG as a viable alternative route for fluid and medication administration during resuscitation and in pre-CPA patients in the prehospital setting as one of emergency treatments authorized for ELSTs.

## Conclusion

The rate of success of IVI in adult CPA patients has been increasing yearly in Japan. However, establishing a peripheral venous line in pediatric patients (1-7 years old) by ELSTs is extremely difficult in prehospital settings. As the study findings indicated IOI with BIG was easy and rapid, we propose to authorize IOI with BIG for ELSTs as an alternative option in case IVI is extremely difficult or impossible in adult and pediatric patients.

## Competing interests

The authors declare that they have no conflict of interest regarding any financial or personal relationships with the manufacturers, or with any other people or organization that could inappropriately influence or bias their work.

## Authors' contributions

Both AH and TN took part in the design and coordination of the study. KI collected the data from the Utstein style database of Japanese FDMA. KI and AH made overviews of the material. KI wrote the first draft of the manuscript and performed the statistical analysis. TN revised it critically for important intellectual content. MT revised the statistical analysis. All authors read and approved the final manuscript.

## Authors' information

KI is an Emergency Life-Saving Technician at the Fire Department, Kyotanabe City, Japan. He is also a research fellow at the Department of Emergency and Critical Care Medicine, Kansai Medical University, Osaka, Japan. TN is an Emergency Physician and Traumatologist, and a Professor at the Department of Emergency and Critical Care Medicine, Kansai Medical University, Osaka, Japan. MT is an Emergency Physician and Traumatologist at the Department of Emergency and Critical Care Medicine, Kansai Medical University, Osaka, Japan. AH is an Emergency Physician and Traumatologist, and Associate Professor at the Department of Emergency and Critical Care Medicine, Fujita Health University, Aichi, Japan.
